# Speed‐dependent locomotor patterns during steady swimming in a demersal shark

**DOI:** 10.1111/jfb.70043

**Published:** 2025-04-03

**Authors:** Fidji Berio, Camille Morerod, Valentina Di Santo

**Affiliations:** ^1^ Department of Zoology Stockholm University Stockholm Sweden; ^2^ Scripps Institution of Oceanography University of California, San Diego La Jolla California USA

**Keywords:** biomechanics, kinematics, locomotion, sharks, speed, swimming

## Abstract

Swimming ability is critical for navigating complex benthic habitats, yet the biomechanical strategies demersal sharks employ to modulate body and fin movements across varying speeds remain largely unexplored. This study examines speed‐dependent kinematic patterns in the small‐spotted catshark (*Scyliorhinus canicula*), a benthic species with limited endurance for sustained swimming. Using high‐speed videography in a flow tank, we quantified adjustments in tail beat frequency, body angle, wave speed and curvature across a range of speeds (0.5–6 body lengths per second). Our results reveal that *S. canicula* exhibits distinct kinematic shifts as speed increases, adopting a more streamlined posture and increasing tail beat frequency to accommodate higher flow rates. Principal component analysis identified swimming speed as the primary factor influencing kinematic variation, with higher speeds necessitating more consistent body alignment and tail movement. Strouhal numbers within the optimal range for propulsive efficiency (0.2–0.4) at intermediate speeds (1–2 BL s^−1^) suggest that *S. canicula* maximizes energetic efficiency within this range, although further research is required to elucidate the metabolic implications. This study establishes a foundational framework for understanding the biomechanics of steady swimming in a demersal shark, providing insights into the ecological and evolutionary pressures shaping locomotor adaptations in benthic species.

## 1 INTRODUCTION

Sharks are a diverse group of apex and mesopredators that have exhibited remarkable resilience in the past hundred million years (Compagno, 1990; Guinot & Cavin, 2016). Shark morphological characteristics reflect the environment they inhabit (Sternes et al., 2024). For example, some sharks have the iconic torpedo‐shaped bodies of pelagic species, such as the great white, and seem perfectly adapted for high‐speed cruising through open waters. Their impressive swimming capacity allows them to dive deeper, move poleward or venture further offshore to effectively adjust their geographic range and avoid hostile conditions (Braun et al., 2023; Coulon, Elliott, et al., 2024; Hammerschlag et al., 2022; Rummer et al., 2022; Townhill et al., 2023). In contrast, benthic species, like carpet sharks, have evolved flattened and elongated bodies, which support a more sedentary and bottom‐dwelling lifestyle (Klimley, 2013; Lauder & Di Santo, 2015). Benthic sharks spend most of their time resting, punctuated by brief bouts of slow, exploratory swimming along the substrate. As a result, they are rarely observed sustaining steady swimming for extended periods. Unsurprisingly, they have not been a common choice for laboratory‐based swimming performance experiments, which typically require prolonged, continuous swimming. Consequently, little is known about their swimming mechanics across a range of speeds. Studying shark locomotion presents significant challenges due to their vast diversity in size and ecological niches, therefore laboratory research is restricted to a few smaller species and early life stages (Graham et al., 1990; Lowe, 1996; Sepulveda et al., 2007; Wilga & Lauder, 2000, 2001). Additionally, discrepancies between volitional swimming and “forced” speed‐controlled flume experiments complicate efforts to generalize findings across species (Bernal et al., 2001; Iliou et al., 2023; Lowe, 1996; Ryan et al., 2015; Webb & Keyes, 1982). These limitations underscore the gaps in our understanding of shark swimming mechanics and highlight the need for broader, more integrative approaches.

Despite these challenges, the literature provides some general patterns of shark swimming. While propulsion is primarily achieved through axial undulation (Maia et al., 2012), pectoral fins play a role in cruising and manoeuvring (Hoffmann et al., 2019; Hoffmann & Porter, 2019; Wilga & Lauder, 2000), and even contribute to creating thrust in species such as angel sharks (Lauder & Di Santo, 2015). Dorsal fins assist in generating thrust and enhancing stability (Lingham‐Soliar, 2005; Maia & Wilga, 2013a, 2013b), while the heterocercal tail is important for generating thrust and lift, and facilitating complex manoeuvres (Lauder, 2000; Wilga & Lauder, 2002). Efficient locomotion relies on the precise coordination of body and fin movements, minimizing energy expenditure. This optimization is crucial for routine activities and sustaining relocations in response to climate change (Vilmar & Di Santo, 2022). While the remarkable migratory abilities of pelagic sharks (e.g., great white sharks [Bonfil et al., 2005, Weng et al., 2007] and basking sharks [Doherty et al., 2017, Skomal et al., 2009]) are well‐documented, how locomotor traits in benthic sharks are modulated during steady swimming remains largely unquantified.

The small‐spotted catshark *Scyliorhinus canicula* L. 1758 is a small demersal shark inhabiting the continental shelf and upper continental slope along the eastern central to northern Atlantic coast and the Mediterranean Sea (Ellis et al., 2009), typically found at depths of up to 800 m (Mytilineou et al., 2005; Rodríguez‐Cabello et al., 2004). This species has been the subject of extensive biological research, making it a model organism to study the evolution and development of gnathostomes (Ballard et al., 1993; Berio et al., 2021; Coolen et al., 2008; Debiais‐Thibaud et al., 2015; Oulion et al., 2011; Zimm et al., 2023). However, detailed swimming kinematics have not been described for this species. A major reason for this gap is that *S. canicula* is not a cruiser and prefers slow swimming speeds and short bouts (West et al., 2023) over long‐distance movements (Rodríguez‐Cabello et al., 2004). These behavioural traits have frustrated experimenters, leading them to conclude that this “relatively inactive” species may be unsuitable for swimming studies in swim tunnels, as the sharks often exploit flow patterns and wall effects to sink and “remain motionless on the bottom” (Bushnell et al., 1989; Butler et al., 1986). Despite the challenges of measuring oxygen consumption in water channels due to their limited swimming endurance, kinematic analyses offer an alternative approach to assessing swimming performance. By examining complete fin beat cycles, which *S. canicula* consistently executes even during short swimming bouts, it is possible to infer how these sharks sustain movement across different speeds.

This study aimed to characterize the swimming kinematics of *S. canicula* across the full range of flow speeds at which it can actively swim in laboratory conditions. By analysing these kinematic patterns, we seek to infer optimal swimming speeds for *S. canicula* and compare these findings with existing data on other shark species. This study provides a quantitative reference on the locomotion of demersal sharks from temperate waters, contributing to the understanding of swimming in benthic species.

## 2 METHODS

### 2.1 Sharks

Juveniles of *S. canicula* (females *n* = 2, males *n* = 3) ranged from 15 to 17 cm (15.8 ± 0.8 cm) in body length (BL, from the tip of the snout to the tip of the tail). The number of specimens (*n* = 5) was chosen based on a power analysis to achieve ≥0.6 power, with medium effect size (Cohen, 2013). Sharks were obtained as embryos from the Station Biologique de Roscoff (Brittany, France) and originated from a North Atlantic population. They hatched and were raised in the facility at Stockholm University under the approved Animal Ethics Protocol (no. 11924‐2020). Water in the tank was kept at 15 ± 1°C and individuals were fed thawed seafood every day, but fasted for 24 h prior to the experiment.

### 2.2 Experimental setup

The experiment was conducted in a 30.75‐L flow tank (Loligo Systems) comprising a 4.5‐L working section (23 cm length × 14 cm width × 14 cm height). We captured synchronized high‐speed videos (1000 FPS; Chronos 2.1, Krontech; 24 mm lens) from two orthogonal cameras (lateral and dorsal views) of catsharks during steady swimming in the working section. A 45° incline was designed to trigger swimming in catsharks facing the incoming flow and was made of honeycomb to ensure streamlined flow as described in previous work (Di Santo & Kenaley, 2016). Swimming experiments were conducted at a controlled temperature of 18°C to ensure consistency in kinematic viscosity (Vogel, 2013) after sharks were adjusted to the flow tank water chemistry and temperature.

The experiment consisted of analysing one tail beat of each catshark during steady swimming at 11 speeds: 0.5, 0.75, 1, 1.25, 1.5, 1.75, 2, 3, 4, 5 and 6 body lengths per second (BL s^−1^). The speeds were tested in randomized order to minimize carry‐over effects (Di Santo et al., 2017). Before each experiment, individuals were measured to the closest millimetre by placing a ruler under the tank. The length of each individual was used to calculate the flow speeds of interest in BL s^−1^. The catshark was subsequently acclimated in the flow tank for 30 min before swimming at the selected flow speeds. The catshark was allowed to recover in the working section at minimum flow speed (< 0.2 BL s^−1^) for at least 5 min before experiencing the next flow speed. We did not process the videos at 0.5, 0.75, 1.75 and 6 BL s^−1^ for one catshark because it was not swimming steadily at those speeds, therefore our final dataset included 51 sequences.

### 2.3 Data extraction

**FIGURE 1:**
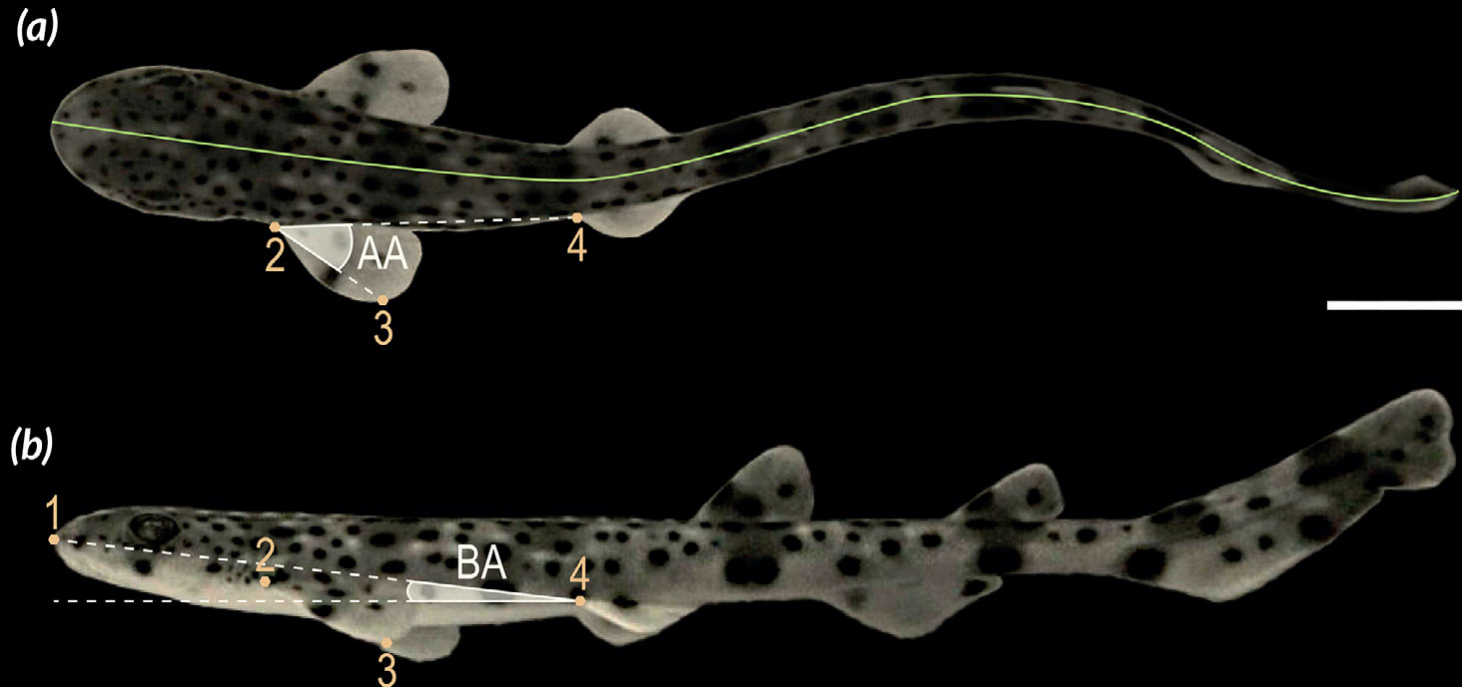
Data retrieved from (a) dorsal and (b) lateral views of *Scyliorhinus canicula* swimming at different speeds. Green, body midline. Gold, landmarks: 1, tip of snout; 2, insertion of left pectoral fin; 3, tip of left pectoral fin; 4, insertion of left pelvic fin. AA, angle of attack in three dimensions; BA, body angle in two dimensions. The scale bar is 1 cm.

We extracted two‐dimensional (2D) and three‐dimensional (3D) spatial coordinates from videos to characterize swimming kinematics during complete tail beat cycles. First, 200 2D coordinates describing the body midline were extracted from dorsal view videos using CurveMapper (Di Santo et al., 2021) in Matlab (vR2015a) (Figure 1). Four 2D landmarks were further digitized with DLTdv8a (v8.2.9) (Hedrick, 2008) on the lateral view (Figure 1b) and three of them (2, 3 and 4) were also set on the corresponding dorsal view to get 3D coordinates (Figure 1a).

### 2.4 Description of swimming variables

The swimming kinematics of catsharks were quantified using 2D and 3D coordinates to capture key aspects of their locomotor dynamics. Tail beat frequency (TBF), representing the number of oscillations per second, and tail amplitude (*A*), the lateral displacement of the tail tip during a complete oscillatory cycle, were measured to describe the fundamental undulatory motions. Wave speed (*c*) and wavelength (*λ*) were calculated to characterize the propagation and spatial periodicity of the travelling wave along the body. Maximum body curvature (*k*_max_), along with its location along the body, provided insights into the degree and distribution of flexibility during propulsion. Detailed methodologies for quantifying these variables are available in Di Santo et al. (2021).

As a biomechanical proxy for energetic costs of swimming, we estimated fin effort, calculated as the product of tail beat frequency and tail amplitude (TBF × *A*) following the approach of Feilich (2017). In addition, angular parameters were examined to understand body posture and fin movements. The three‐dimensional angle of attack (AA) was measured between the tip of the left pectoral fin and the insertion of the left pelvic fin, providing a metric for the fin orientation relative to the surrounding flow (Figure 1a). From lateral view coordinates, the body angle (BA) was determined using the positions of the snout tip and the pelvic fin insertion (landmarks 1 and 4, respectively, in Figure 1b), capturing the posture of the body during swimming.

To contextualize these kinematics within hydrodynamic and energetic frameworks, two non‐dimensional parameters were calculated. The Reynolds number (Re) was defined as:

Re=U×BLν,

where *U* is the swimming speed in m s^−1^, BL is the body length in m and *ν* is the kinematic viscosity of water of 1 × 10^−6^ m^2^ s^−1^ (Cohen & Boyle, 2010). The Reynolds number reflects the fluid regime encountered by the catsharks. The Strouhal number (St), an indicator of swimming efficiency, was calculated as:

$$\mathrm{St}=\frac{\mathrm{TBF}\times A}{U},$$

where TBF is the tail beat frequency in s^−1^, *A* is the tail amplitude in m and *U* is the swimming speed in m s^−1^ (Triantafyllou et al., 1991). The Strouhal number describes the relationship between oscillatory propulsion and forward motion. Previous hydrodynamic studies have shown that efficient propulsion in undulatory swimmers occurs within the optimal Strouhal range of 0.2–0.4 (Taylor et al., 2003; Triantafyllou et al., 1991), which corresponds to the most effective vortex shedding, where thrust production is maximized and energy loss due to excessive wake turbulence is minimized. When St is too low (< 0.2), propulsion efficiency decreases because the tail beats are too slow relative to forward speed, leading to weak vortex formation and reduced thrust. Conversely, when St is too high (> 0.4), excessive tail beating creates unsteady wake patterns, increasing drag and reducing net efficiency. Together, these variables provide an assessment of the kinematics and hydrodynamics underpinning catshark locomotion.

### 2.5 Data analysis

The fin effort, angle of attack and body angle were computed with our custom code and the remaining swimming variables were computed with the R code published by Goerig et al. (2021). First, we performed a principal component analysis (PCA) on independent swimming variables to visualize the structure of *S. canicula* swimming characteristics by speed and the grouping of variables. Then, for each kinematics variable, we tested for the homogeneity of variances with Levene's tests and data normality with Shapiro–Wilk tests. When both tests did not reject H0, we subsequently looked for differences across speeds using repeated measures analysis of variance (ANOVA) models eventually followed by pairwise post‐hoc comparisons with Tukey adjustment. When conditions of homoscedasticity or normality were not met, we used alignment ranks transformation ANOVA (ART ANOVA) eventually followed by post hoc contrast tests with Tukey adjustment. Data pre‐processing, statistical tests and renderings were performed with R (v4.2.1) (R Core Team, 2022). In addition to R base packages for statistics, we used the emmeans package (v1.8.3) (Lenth, 2022) for pairwise comparisons and the ARTool package (v0.11.1) (Kay et al., 2021) for ART ANOVAs. The significance threshold for statistical tests was set to 0.05.

## 3 RESULTS

**FIGURE 2:**
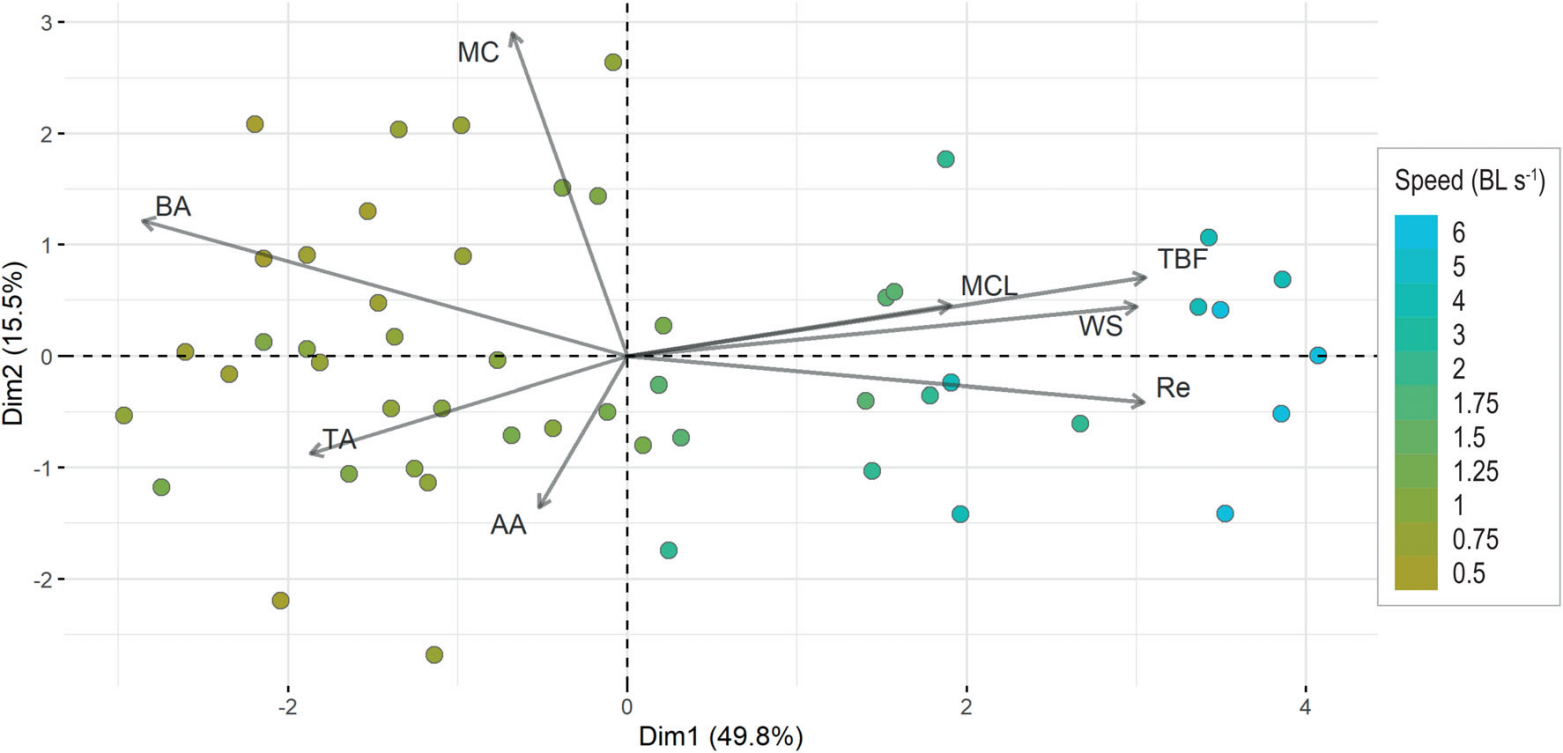
Projection of descriptive variables of *Scyliorhinus canicula* swimming at different speeds on the two principal axes of a principal component analysis. The main axis of variation relates to swimming speed and is primarily driven by body angle, tail beat frequency, Reynolds number and wave speed, while the second axis of variation is shaped by body curvature, angle of attack and body angle. AA, angle of attack; BA, body angle; TA, tail amplitude; MC, maximum body curvature; MCL, location of maximum curvature along the body; Re, Reynolds number; TBF, tail beat frequency; WS, wave speed. BL, body length.

Sharks adjust both their body posture and tail fin movements as they increase swimming speed. Specifically, they reduce their body angle, adopting a more horizontal position, while simultaneously increasing their tail beat frequency. The PCA analysis showed that the first two axes contain 65.3% of the total variance and the main axis relates to *S. canicula* swimming speed (Figure 2), suggesting that speed is the main driver in the changes in posture and kinematics observed in this shark. In particular, four variables contribute the most to the main axis (contribution > 10%) and are structured into two groups: body angle on the one hand (18.7% contribution to the main axis) and tail beat frequency, Reynolds number and wave speed (21.4%, 21.2% and 20.7% contribution to the main axis, respectively) on the other hand.

Furthermore, variables within those two groups are negatively correlated, meaning, for example, that *S. canicula* displays a lower body angle at high tail beat frequencies, which also occurs as swimming speed increases (Figure 2).

The variables contributing the most to the second axis are the maximum body curvature, the angle of attack and the body angle (62.0%, 13.6% and 10.9%, respectively). In addition, the maximum body curvature and the angle of attack are negatively correlated with each other (Figure 2).

**FIGURE 3:**
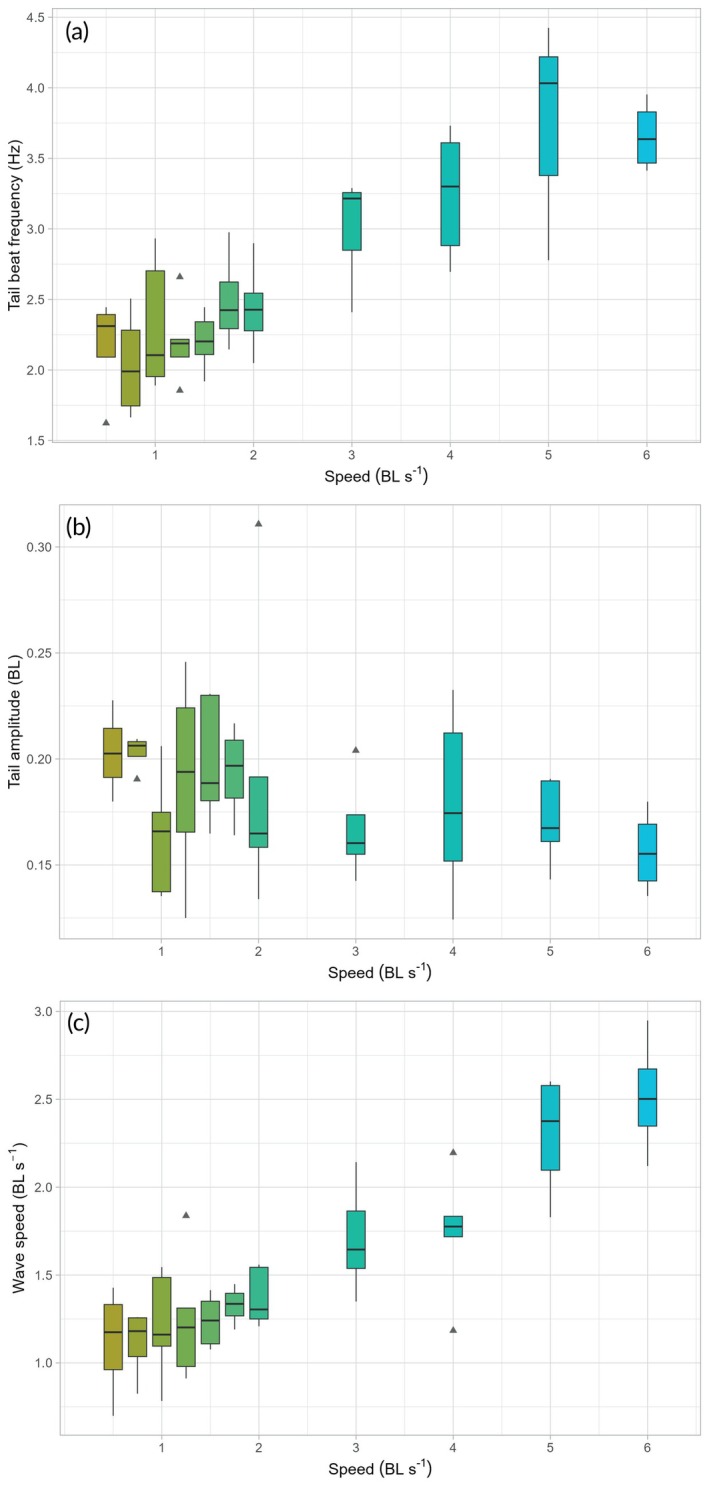
Swimming kinematics in *Scyliorhinus canicula* swimming across 11 speeds. (a) Tail beat frequency is similar across speeds from 0.5 to 2 BL s^−1^ and increases linearly beyond 2 BL s^−1^. (b) Tail amplitude is variable and shows no general trend across speeds. (c) Wave speed follows a similar pattern as tail beat frequency but increases by steps, from 2 to 3 BL s^−1^ and 4 to 5 BL s^−1^. On each box, the horizontal line represents the median, whisker lines show the location of the minimum and maximum values, and triangles are outliers. BL, body length.

Across speeds, we found significant differences in tail beat frequency (repeated measures ANOVA, *F*_(10,36)_ = 11.51, *p* < 0.001) and wave speed (repeated measures ANOVA, *F*_(10,36)_ = 13.63, *p* < 0.001), with both variables displaying similar values across 0.5 to 2 BL s^−1^ (Figure 3a,c and Table 1).

**TABLE 1:** Kinematics with post hoc differences across speeds.

Speed (BL s^−1^)	TBF (Hz)	Wave speed (BL s^−1^)	Fin effort (BL s^−1^)	Body angle (degrees)
0.5	2.17 ± 0.38 (a, b)	1.12 ± 0.32 (a)	0.44 ± 0.11 (a, b)	39.4 ± 11.5 (a)
0.75	2.04 ± 0.39 (a)	1.11 ± 0.20 (a)	0.41 ± 0.08 (a,b)	44.6 ± 2.15 (a)
1	2.32 ± 0.47 (a,b)	1.21 ± 0.31 (a,b)	0.37 ± 0.02 (a)	39.6 ± 9.95 (a,b)
1.25	2.20 ± 0.29 (a,b)	1.25 ± 0.37 (a,b)	0.41 ± 0.07 (a)	41.9 ± 3.18 (a)
1.5	2.20 ± 0.20 (a,b)	1.24 ± 0.15 (a,b)	0.44 ± 0.08 (a,b)	39.0 ± 3.41 (a,b)
1.75	2.49 ± 0.35 (a,b,c)	1.33 ± 0.11 (a,b)	0.48 ± 0.07 (a,b,c)	39.0 ± 4.53 (a,b)
2	2.44 ± 0.32 (a,b,c)	1.37 ± 0.17 (a,b)	0.46 ± 0.12 (a,b)	34.8 ± 3.47 (a,b,c)
3	3.00 ± 0.38 (b,c,d)	1.71 ± 0.31 (b)	0.50 ± 0.02 (a,b,c)	28.5 ± 3.99 (b,c,d)
4	3.24 ± 0.45 (c,d)	1.74 ± 0.36 (b,c)	0.57 ± 0.08 (b,c)	23.5 ± 3.22 (c,d)
5	3.77 ± 0.68 (d)	2.32 ± 0.34 (c,d)	0.63 ± 0.07 (c)	19.2 ± 1.51 (c,d)
6	3.66 ± 0.25 (d)	2.52 ± 0.35 (d)	0.57 ± 0.04 (b,c)	14.0 ± 3.34 (d)

*Note*: Results are reported for each speed as mean  ±  standard deviation. Different letters represent a significant difference across speeds.

Abbreviations: BL, body length; TBF, tail beat frequency.

**FIGURE 4:**
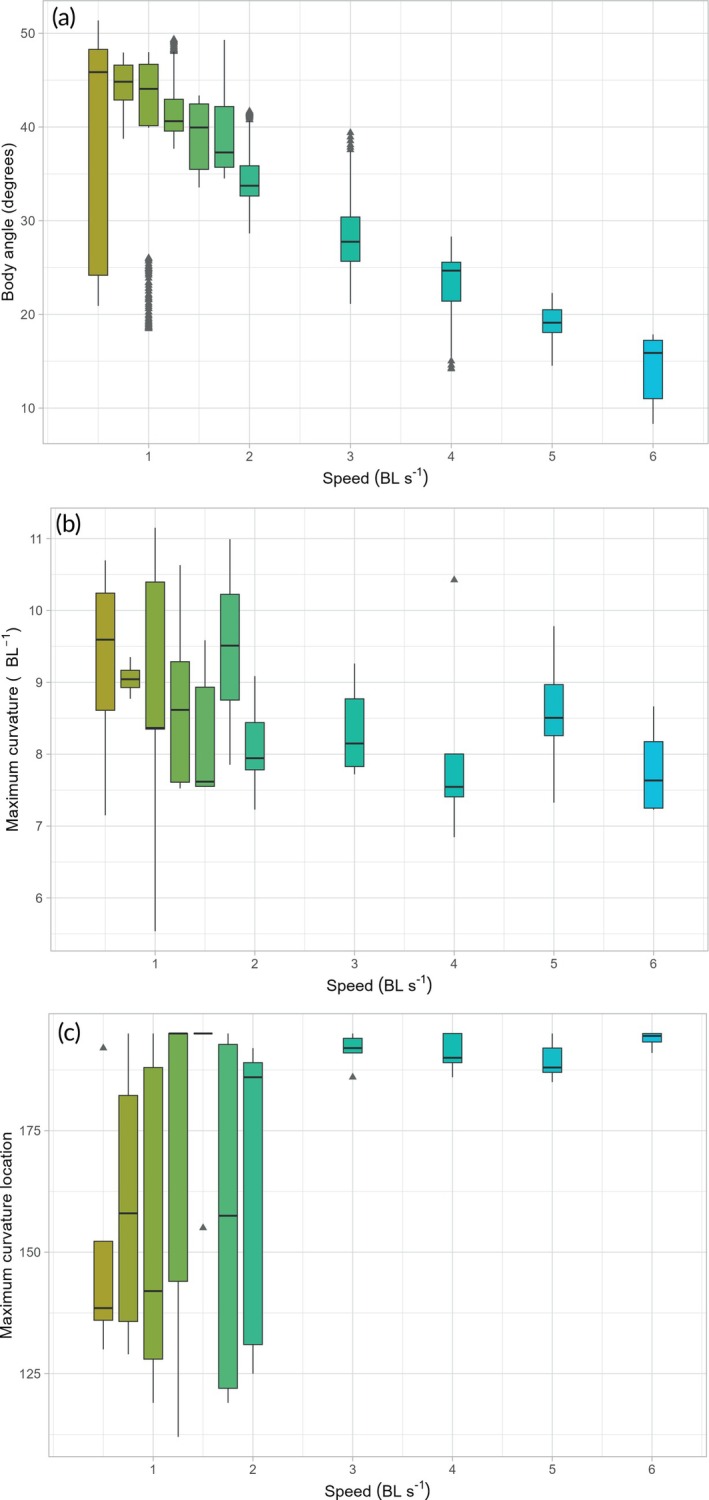
Swimming kinematics in *Scyliorhinus canicula* swimming across 11 speeds. (a) Body angle is highly variable at 0.5 and 1 BL s^−1^ and decreases linearly with speed. (b) Maximum body curvature is variable and shows no general trend across speeds. (c) Location of maximum curvature along the body is highly variable across speeds from 0.5 to 2 BL s^−1^ but restricted to tail tip at 3 BL s^−1^ and beyond. On each box, the horizontal line represents the median, whisker lines show the location of the minimum and maximum values, and triangles are outliers. BL, body length.

We reported no statistical differences in tail amplitude (ART ANOVA, *F*_(10,36)_ = 1.76, *p* = 0.104; Figure 3b) and maximum body curvature (repeated measures ANOVA, *F*_(10,36)_ = 0.92, *p* = 0.527; Figure 4b) across speeds.

**FIGURE 5:**
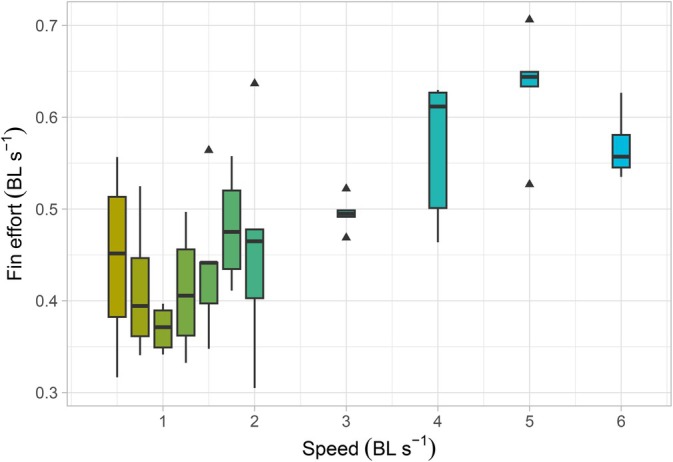
Fin effort (tail beat frequency × tail amplitude) across swimming speeds in *Scyliorhinus canicula* showing a minimum value at 1 BL s^−1^. On each box, the horizontal line represents the median, whisker lines show the location of the minimum and maximum values, and triangles are outliers. BL, body length.

Furthermore, the location of maximum body curvature was not significantly impacted by speed (repeated measures ANOVA, *F*_(10,36)_ = 1.88, *p* = 0.080). Nevertheless, we highlight the high variation of maximum curvature location along the body between 0.5 and 2 BL s^−1^ (except for 1.5 BL s^−1^), while it is restricted to the very tip of the tail when catsharks swim at 3 BL s^−1^ and faster (Figure 4c). Body angle decreases with speed (ART ANOVA, *F*_(10,36)_ = 12.48, *p* < 0.001), with a high variation of values at 0.5 and 1 BL s^−1^ (Figure 4a and Table 1). Fin effort was also statistically different across speeds (repeated measures ANOVA, *F*_(10,36)_ = 7.13, *p* < 0.001) with a minimum around 1 BL s^−1^ (Figure 5). Finally, we found no significant differences in wavelength (repeated measures ANOVA, *F*_(10,36)_ = 1.56, *p* = 0.159) and angle of attack (repeated measures ANOVA, *F*_(10,36)_ = 0.89, *p* = 0.555) across speeds.

We also report that tail beat frequency increases when swimming at speeds beyond 2 BL s^−1^. In contrast, wave speed increases in two distinct phases: first between 2 and 3 BL s^−1^ and then between 4 and 5 BL s^−1^ .

**FIGURE 6:**
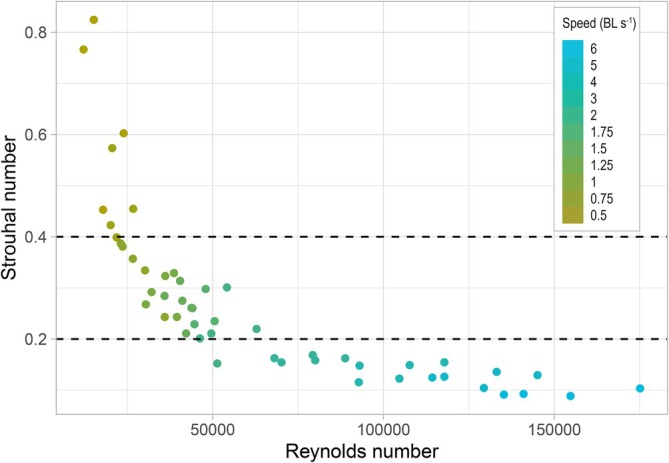
Strouhal number as a function of the Reynolds number, with an indication of the swimming speed in body length per second in *Scyliorhinus canicula*. The optimal range for propulsive efficiency is defined by dashed lines and corresponds to swimming speeds from 1 to 2 BL s^−1^. BL, body length.

Strouhal numbers within the optimal range for propulsive efficiency (0.2 < St < 0.4) correspond to swimming speeds of 1–2 BL s^−1^ in *S. canicula* (Figure 6) (Taylor et al., 2003; Triantafyllou et al., 1991). This result indicates that these intermediate speeds are the most efficient for minimizing energy expenditure while maximizing propulsion for this species.

## 4 DISCUSSION

Quantifying the swimming kinematics of benthic sharks, such as *Scyliorhinus canicula*, is essential for understanding how these species navigate structurally complex habitats while maintaining stability and manoeuvrability (Di Santo & Goerig, 2025). Unlike pelagic sharks, which are adapted for efficient, sustained cruising, benthic sharks exhibit slower, less regular swimming bouts that reflect the demands of their demersal lifestyles. While these traits are ecologically significant, they also pose challenges for studying locomotion under controlled laboratory conditions, where steady locomotion is required (Butler et al., 1986; West et al., 2023). To address these challenges, we employed a swim tunnel with a honeycomb ramp to minimize flow disturbances and facilitate steady swimming, an approach described by previous studies on benthic batoids (Di Santo et al., 2017; Di Santo & Kenaley, 2016). This methodology allowed us to obtain high‐resolution kinematic data across a wide range of swimming speeds, offering new insights into the biomechanical strategies underlying benthic locomotion.

Nevertheless, it should be noted that the challenge of having *S. canicula* swimming steadily has limited our sample size (*n* = 5) and the use of a single tail beat to characterize the kinematics of each specimen at each speed. Using a single complete cycle of movement during steady swimming has, however, already proven relevant to describe a fish's swimming kinematics (Di Santo et al., 2017). When possible, analysing multiple tail beat cycles per fish and speed would be beneficial to refine the amount of intraspecific variation in kinematics values.

Our experiments revealed that *S. canicula* swims across a range of speeds from 0.5 to 6 BL s^−1^, exhibiting distinct kinematic adjustments as speed increases. At the lowest speeds (0.5–0.75 BL s^−1^), individuals struggled to maintain position in the water column, frequently trying to rest on the bottom. This struggle likely reflects the species' reliance on benthic interactions, which are known to reduce energy expenditure through hydrodynamic effects such as drag reduction and lift enhancement (Gerstner & Webb, 1998; Triantafyllou et al., 2000). Since our experimental design required sharks to swim off the substrate, the lack of these benefits may have influenced kinematics at low speeds (Di Santo et al., 2017). Despite the observed preference for slow swimming in natural conditions (West et al., 2023), our results indicate that *S. canicula* achieves its most efficient swimming at intermediate speeds (1–2 BL s^−1^). Within this range, Strouhal numbers fell within the optimal range for propulsive efficiency (0.2 < St <0.4) (Taylor et al., 2003, Triantafyllou et al., 1991). These findings suggest that while *S. canicula* frequently operates at low speeds in the wild, its biomechanical efficiency for steady swimming peaks within this intermediate range, similar to patterns observed in other undulatory swimmers (Di Santo et al., 2017; Di Santo & Goerig, 2025; Wilga & Lauder, 2000).

At speeds below 2 BL s^−1^, we observed high variation in kinematic patterns, likely due to reduced hydrodynamic stability. Insufficient thrust and lift generation at these speeds may make maintaining a steady posture more challenging, resulting in increased variation. Conversely, at higher speeds (> 3 BL s^−1^), biomechanical precision becomes increasingly important, as greater hydrodynamic forces necessitate controlled body postures to minimize resistance (Di Santo et al., 2021; Webb & Keyes, 1982). The upper speed limit for steady swimming in juvenile *S. canicula* appears to be 6 BL s^−1^, at least in laboratory settings.

Comparing our results to previous work highlights a key distinction between preferred and optimal swimming speeds. West et al. (2023) reported that *S. canicula* typically swims at speeds below 0.54 BL s^−1^ in voluntary conditions, with a median of 0.38 BL s^−1^. By contrast, our study identified 1–2 BL s^−1^ as the most efficient range for minimizing energy expenditure per unit distance travelled. Preferred swimming speeds reflect an individual's immediate behavioural and ecological demands, while optimal speeds, typically determined through respirometry, are those that minimize oxygen consumption per unit distance travelled (Tudorache et al., 2013). Since *S. canicula* cannot sustain prolonged steady swimming, making direct metabolic measurements impractical, we inferred its optimal speeds using kinematic proxies such as tail beat frequency and fin effort. A similar approach in the little skate (*Leucoraja erinacea*) identified minimal body angles and metabolic rates at intermediate speeds, reinforcing the idea that optimal performance does not necessarily occur at the lowest speeds a species can achieve (Di Santo et al., 2017). Our results suggest that *S. canicula* follows a comparable pattern, with kinematic efficiency peaking within the 1–2 BL s^−1^ range. However, swimming performance varies widely among shark species, often reflecting ecological and morphological adaptations. For example, species such as blacktip reef sharks (*Carcharhinus melanopterus*) and bonnethead sharks (*Sphyrna tiburo*) achieve optimal Strouhal numbers at slower speeds (closer to 0.8 BL s^−1^) (Eloy, 2012; Kajiura et al., 2022), reflecting adaptations for continuous swimming in the water column. In contrast, kinematic efficiency peaking at higher speeds in *S. canicula* aligns with a benthic lifestyle characterized by alternating short swimming bouts and resting on the bottom. Furthermore, high variation in tail beat frequency and other kinematic parameters at low speeds, as observed in *S. canicula*, has also been reported in other benthic and slow‐swimming species, such as the Caribbean reef shark (*C. perezi*) (Kajiura et al., 2022). By contrast, obligate ram ventilators like the blacktip shark (*C. limbatus*) exhibit strong correlations between tail beat frequency, amplitude and swimming velocity (Kajiura et al., 2022). These differences reflect the distinct biomechanical and morphological strategies sharks use to meet the demands of their respective habitats.

Our findings also highlight the potential limitations of swim tunnel studies in capturing natural behaviour. While controlled experiments allow precise quantification of kinematics, they do not fully replicate the complexities of free‐swimming conditions. Spatial constraints can induce gait transitions at lower speeds than observed in the wild, and fish often reach higher critical swimming speeds in larger flumes or raceways (Kern et al., 2018; Tudorache et al., 2007). Integrating laboratory and field studies, such as using bio‐loggers and large‐scale flumes in natural settings, will be crucial for bridging these gaps (Gallagher et al., 2021; Payne et al., 2015).

Steady swimming performance is closely linked to ecological function, influencing behaviours such as foraging, predator avoidance and habitat selection (Ryan et al., 2015; Walker & Westneat, 2002; Weihs, 1977). Many shark species undertake seasonal migrations driven by reproductive and foraging needs, with pelagic species covering vast distances (Bonfil et al., 2005; de la Parra Venegas et al., 2011). While benthic sharks like *S. canicula* are less mobile, our findings suggest they can sustain efficient swimming at speeds suitable for small‐scale movements, such as relocating within their home range.

In the face of climate change, locomotor capacity may play a crucial role in species' ability to cope with environmental shifts. Highly mobile sharks can migrate to locate suitable habitats, but species with strong site fidelity, like *S. canicula* (Rodríguez‐Cabello et al., 2004), may rely more on physiological tolerance to changing conditions (Coulon, Elliott, et al., 2024; Di Santo, 2024). Nevertheless, extreme environmental stressors, such as warming and ocean acidification, can still disrupt normal swimming behaviour, potentially impairing foraging efficiency and predator evasion (Coulon, Pilet, et al., 2024; Di Santo, 2015, 2016; Green & Jutfelt, 2014). Understanding how swimming mechanics interact with these environmental factors will be essential for predicting the resilience of benthic sharks under future ocean conditions (Di Santo, 2022; Vilmar & Di Santo, 2022).

This study provides the first comprehensive analysis of speed‐dependent kinematics in *S. canicula*, revealing that while this species prefers slow swimming in the wild, its most efficient swimming occurs at 1–2 BL s^−1^. This optimal range aligns with broader patterns observed in undulatory swimmers and highlights the importance of considering both preferred and optimal speeds when assessing locomotor performance. Future research integrating kinematic, metabolic and ecological data will be critical for advancing our understanding of benthic shark locomotion and its role in shaping species resilience in a changing ocean.

## 5 CONCLUSIONS

This study quantifies the swimming kinematics of *Scyliorhinus canicula*, providing new insights into the biomechanical strategies that underpin benthic shark locomotion. Our findings reveal distinct speed‐dependent adjustments in body posture and tail movements, highlighting the challenges *S. canicula* faces in maintaining stability at low speeds. Using kinematic proxies, we infer that this species achieves its most efficient swimming at intermediate speeds, aligning with broader patterns observed in fish locomotion (Di Santo et al., 2017).

Future research integrating kinematic analyses with muscle activity monitoring, energetics and hydrodynamic modelling could further elucidate the energetic trade‐offs between benthic and pelagic locomotion (Jayne & Lauder, 1995). Investigating how environmental stressors, such as rising temperatures and ocean acidification, affect swimming performance would provide critical insights into the resilience of *S. canicula* to climate change. Additionally, exploring ontogenetic shifts in fin morphology, body flexibility and predator–prey dynamics could refine our understanding of how locomotor capacity changes and influences the ecological roles of demersal sharks in ecosystems.

By linking biomechanics to ecological function and environmental pressures, this study underscores the importance of locomotor performance in shaping survival strategies and ecological interactions in a changing ocean (Di Santo, 2022; Vilmar & Di Santo, 2022). These findings provide a foundation for future research into the complex interplay between physiology, behaviour and environmental adaptation in benthic elasmobranchs.

## AUTHOR CONTRIBUTIONS

F.B.: Project administration, swim tunnel experiments, writing of the original draft, reviewing and editing the draft, data analysis, interpretation and visualization. C.M.: Swim tunnel experiments, reviewing and editing the draft. V.D.S.: Project administration, reviewing and editing the draft, data interpretation, funding, equipment and tools.

## FUNDING INFORMATION

This research was supported by the Human Frontier Science Program (award no. RGP 0010/2022) to Valentina Di Santo.

## CONFLICT OF INTEREST STATEMENT

The authors declare no potential conflict of interests.

## Supporting information


**Data S1.** Supporting Information.

## Data Availability

The data that support the findings of this study are openly available in GitHub at https://github.com/fberio/scanicula_steady_swimming/.
